# A Case of Nephritis—Lumbo-Renal Abscess—Death—Postmortem Examination

**Published:** 1849-08

**Authors:** E. B. Haskins

**Affiliations:** Clarksville, Tenn.


Art. III.—*A Case of Nephritis.—Lumbo-renal Abscess.—Death.—Post-Mortem Examination.* By Dr. E. B. Haskins, of Clarksville, Tenn.

Mr. J—— S——, apothecary, aet. 25, of small stature, and delicate habit, was attacked early in the month of August, 1848, with slight uneasiness in the right side, causing him to bend his body in that direction. In a few days he was seized, after taking a cold bath, with fever, attended with considerable increase of the local uneasiness. I was called to him on the 19th of August. He then had full pulse, beating 110 times in a minute, anorexia and foul tongue; decubitus, mostly on right side; pain and tenderness on pressure immediately over the right kidney; pain and retraction of right testicle; frequent desire to urinate; urine normal in appearance and quantity.

The seat of the pain and tenderness, together with the pain and retraction of the testicle, and the frequent desire to micturate, all pointed to the kidney as the suffering organ; whilst the absence in the urine of albumen and other products of disordered secretion, indicated with certainly equal probability that the inflammation did not involve the proper glandular structure of the kidney, but rather the investing tunic or capsule of that organ, together with the cellular structure, and possibly the muscles upon which the organ rests, constituting the *perinephritis* of M. Rayer.

The treatment was actively antiphlogistic; embracing cups to the loins, purgatives and nauseants; to which was added colchicum, under the belief that the disease might be of a rheumatic character. About this time the urine was carefully analyzed without anything abnormal being detected.

The disease continued acute for about six weeks, towards the latter portion of which time, the active antiphlogistic treatment was abandoned for mercury in alterative doses, with tinct. of iodine freely applied with a shaving brush, over the region of tenderness and pain.

When the acute symptoms had fairly subsided, the urine, on being allowed to stand, precipitated considerable quantities of uric acid, and the urates of soda and ammonia, There appeared, coetaneously with this, a free warm diaphoresis, which, while it appeared likely to effect a change favorable to the patient, strengthened the supposition of the rheumatic character of the inflammation.

The length of time that elapsed before suppuration occurred, together with the pathological condition of the heart, as revealed at the necroscopy, also give countenance to this element of the diagnosis.

The amendment, however, was only partial. The tongue assumed a more healthy appearance, the appetite slightly improved, and the pulse lessened in frequency and force. The sub-acute fever still continued, with the red and fawn colored amorphous deposites in the urine.

This condition of things continued for two weeks when slight rigors came on, followed by high fever, presenting more or less regular diurnal paroxysms. The urine again assuming a healthy appearance, tonics, with light nutritious diet, were ordered, to sustain the system under what was thought to be purulent formation. The local treatment consisted in the use of camphorated mercurial ointment, ointment of iodine, and, occasionally, the application of warm poultices, over the seat of pain and tenderness.

The condition of the patient and the main features of the treatment were materially the same up to the middle of December, when he passed out of my hands, at which time he was able (as, indeed, he had been ever since the decline of the acute stage,) to sit up in bed, walk about the room, and, in good weather, even to take exercise in a carriage.

I saw the patient no more, professionally, until the last of February, when I met, in consultation upon his case, Drs. Wilcox, Cooper, Donoho, and Finley.

We learned from the then attending physician (Dr. W.) that the treatment had consisted mainly in an active course of mercury ; that salivation was induced ; that the patient improved for a time, but had grown worse from imprudence, as was thought, in over-exercise.

The condition of the patient at this time was as follows : Considerable emaciation, with an anemic aspect of body ; yellow skin ; face tumid ; pulse 120 ; stomach and bowels irritable ; but little appetite ; thirst ; tumefaction, with some tenderness over right kidney.

The physical signs were : Chest resonant on percussion; respiratory murmur feeble but somewhat rude; a bellows-murmur (of anemia?) heard on the first sound of the heart.

The treatment agreed upon consisted of tonics, with light animal diet, &c., but proved of no avail. The patient gradually sunk and finally died on the 12th of March, 1849.

*Autopsy, twelve hours after death.*—Body emaciated; skin of a pale yellowish hue; on right side, crest of ilium overlapped the false ribs. Thorax—on the left side of the mediastinum, about sixteen ounces of a dark yellowish serosity, that gave, with nitric acid, the characteristic reaction of bile,* occupied the posterior portion of the chest; feeble, but extensive adhesions between the costal and pulmonary pleuras of the right side, as well as adhesions of the lobes of the right lung. The apices of both lungs were marbled by a considerable deposit of melanotic matter. The lining membrane of the larger bronchial tubes was red, and covered with a viscid mucus. The pericardium contained three or four ounces of a fluid that escaped without examination; the cardiac texture softened, the fingers piercing the walls of the heart with great ease.† Abdomen—Liver slightly enlarged, but texture healthy in appearance. Gall-bladder distended with dark thick bile, and contained four gall-stones of the size of a grain of coffee; spleen greatly enlarged. The mucous coats of the stomach and bowels healthy, as far as examined. Mesenteric glands normal. The left kidney

---

* From this fact, we would presume that the jaundiced hue of the skin depended in this case upon an excess of *biliphæin*, and not that of *hæmaphæin*, as supposed by Golding Bird to be the case, in the yellow skin of chlorotic and anemic subjects, generally.

† No symptom drew special attention to this organ; and, therefore, it was not examined until late in the disease;—yet it is quite probable that pericarditis supervened in the course of the malady.

was sound; right kidney enlarged, but presented no decided marks of alteration of structure, except considerable thickening of the investing capsule. Between the right kidney and upper portion of the psoas magnus and quadratus lumborum muscles, embracing the posterior face of the investing tunic of the kidney, the adipose tissue beneath, and the anterior face of the muscles above named, an abscess, lined by a dense cyst, was found, containing about ten ounces of a whitish curdy-looking pus. The circumference of the abscess corresponded exactly with that of the kidney.

Clarksville, Tenn., July 3, 1849.

